# *Evi1* Counteracts Anti-Leukemic and Stem Cell Inhibitory Effects of *All-Trans* Retinoic Acid on *Flt3*-ITD/*Npm1c*-Driven Acute Myeloid Leukemia Cells

**DOI:** 10.3390/biomedicines8100385

**Published:** 2020-09-28

**Authors:** Chi Huu Nguyen, Alexander M. Grandits, George S. Vassiliou, Philipp B. Staber, Gerwin Heller, Rotraud Wieser

**Affiliations:** 1 Division of Oncology, Department of Medicine I, Medical University of Vienna, 1090 Vienna, Austria; chi.nguyen@x4pharma.com (C.H.N.); alexander.grandits@meduniwien.ac.at (A.M.G.); gerwin.heller@meduniwien.ac.at (G.H.); 2 Comprehensive Cancer Center, 1090 Vienna, Austria; 3 Wellcome Medical Research Council Cambridge Stem Cell Institute, Department of Haematology, University of Cambridge, Cambridge CB2 0AW, UK; george.vassiliou@sanger.ac.uk; 4 Division of Hematology and Hemostaseology, Department of Medicine I, Medical University of Vienna, 1090 Vienna, Austria; philipp.staber@meduniwien.ac.at

**Keywords:** AML, leukemia stem cells, *all-trans* retinoic acid, *FLT3*-ITD, *EVI1*, *MECOM*

## Abstract

*All-trans* retinoic acid (atRA) has a dramatic impact on the survival of patients with acute promyelocytic leukemia, but its therapeutic value in other types of acute myeloid leukemia (AML) has so far remained unclear. Given that AML is a stem cell-driven disease, recent studies have addressed the effects of atRA on leukemic stem cells (LSCs). atRA promoted stemness of *MLL-AF9*-driven AML in an *Evi1*-dependent manner but had the opposite effect in *Flt3*-ITD/*Nup98-Hoxd13*-driven AML. Overexpression of the stem cell-associated transcription factor *EVI1* predicts a poor prognosis in AML, and is observed in different genetic subtypes, including cytogenetically normal AML. Here, we therefore investigated the effects of *Evi1* in a mouse model for cytogenetically normal AML, which rests on the combined activity of *Flt3*-ITD and *Npm1c* mutations. Experimental expression of *Evi1* on this background strongly promoted disease aggressiveness. atRA inhibited leukemia cell viability and stem cell-related properties, and these effects were counteracted by overexpression of *Evi1*. These data further underscore the complexity of the responsiveness of AML LSCs to atRA and point out the need for additional investigations which may lay a foundation for a precision medicine-based use of retinoids in AML.

## 1. Introduction

Acute myeloid leukemia (AML) is an aggressive hematopoietic malignancy whose incidence increases with age [1,2]. Even though the number of genetic lesions per individual is low compared to other cancers [3], its genetic causes are complex and heterogeneous [4,5,6,7,8]. A number of recurrent chromosome rearrangements and point mutations have been described, among them the translocation t(8;21) which gives rise to the *acute myeloid leukemia 1*—*eight-twentyone* (*AML1-ETO*) fusion gene, 11q23 rearrangements involving the *mixed lineage leukemia* (*MLL*) gene, *nucleoporin 98* (*NUP98*) fusions, *nucleophosmin 1* (*NPM1*) mutations, and *fms related receptor tyrosine kinase 3* internal tandem duplications (*FLT3*-ITD) [4,5,6,9,10]. Further, aberrant expression of genes, including that encoding the transcription factor Ecotropic Viral Integration site 1 (EVI1), is a typical feature of AML [4,7,11,12,13]. Many molecular and genetic aberrations have prognostic value and/or represent potential or actual targets for rationally designed therapeutics [4,5,6,7,12,13,14]. Indeed, several targeted drugs were recently approved for use in AML and will complement standard chemotherapy for selected groups of patients [14]. However, acute promyelocytic leukemia (APL), characterized by rearrangements of the *retinoic acid receptor alpha* (*RARA*) gene, has benefited more than any other AML subtype from targeted therapies: addition of the RARA ligand *all-trans* retinoic acid (atRA) to its therapy has greatly improved APL patient survival for the last few decades [15,16,17]. Despite the striking success of atRA in APL, and even though atRA also causes blast differentiation and sensitization to chemotherapy in other types of AML in vitro [18,19,20,21,22,23,24,25,26,27], clinical benefit of atRA in non-APL AML has not been consistently demonstrated so far [20,27,28,29,30,31]. Further, attempts to identify genetically defined subgroups of patients that may respond to atRA-containing therapy have yielded contradictory results [20,27,29,30,31,32].

Both normal and leukemic hematopoiesis are organized in a hierarchical manner, and emanate from mostly quiescent stem cells (hematopoietic stem cells, HSCs, or leukemic stem cells, LSCs, respectively) that reside in a specialized niche in the bone marrow (BM) [33,34,35]. These stem cells give rise to highly proliferative progenitors, which in normal hematopoiesis differentiate into non-dividing functional blood cells, but in malignant hematopoiesis form the bulk of the only partially differentiated leukemic cell mass [33,34,35]. LSCs play key roles not only in leukemia emergence, but also in chemotherapy resistance and relapse [33]. Therefore, determining the effect of a potential therapeutic on LSCs may be pivotal to understanding its clinical effectiveness [33]. Some recent studies investigated the impact of atRA on AML LSCs, with divergent results [27]. atRA inhibited stem cell abundance and activity in a mouse model of AML driven by a *Nup98-Hoxd13* fusion gene together with an *FLT3*-ITD [36]. By contrast, atRA promoted serial replating ability—considered as a readout of stem cell activity—in *AML1-ETO*-expressing murine BM cells [21]. Similarly, in an *MLL-AF9*-driven mouse model of AML, atRA augmented stem cell abundance, quiescence, and activity in a manner that was dependent on the expression of *Evi1* [37]. *EVI1* and atRA also collaborated to promote stem cell-related properties in human AML cell lines and primary samples [37].

In the present study, we asked whether *EVI1* would also interact with atRA to alter LSC-related properties on the background of different genetic driver lesions. Since 21% of *EVI1*-overexpressing AMLs are cytogenetically normal [13], we sought to employ a model for cytogenetically normal AML. The most frequent mutations in cytogenetically normal AML affect the *FLT3* and *NPM1* genes (the latter lead to a predominantly cytoplasmic localization of the chaperone protein NPM1, hence are referred to as “*NPM1c*”) [4,5,6], and mice whose hematopoietic cells carry a *Flt3*-ITD and an *Npm1c* allele develop an aggressive, AML-like disease [38]. The *Flt3*-ITD/*Npm1c* model was therefore used in the current study. We found that atRA inhibited leukemia cell (LC) viability as well as LSC-related properties in Evi1^low^ *Flt3*-ITD/*Npm1c*-driven AML, but these effects were counteracted by experimental *Evi1* expression.

## 2. Experimental Section

### 2.1. Ethics Approval

Animal experiments were approved by the Animal Ethics Committee of the Medical University of Vienna and the Austrian Federal Ministry of Education, Science, and Research (GZ66.009/0309-WF/V/3b/2015, 3 November 2015). Federation of European Laboratory Animal Science Associations and Austrian guidelines to minimize animal distress and suffering were followed.

### 2.2. Ex Vivo Culture of Cells from Flt3-ITD/Npm1c-Driven Murine AML and Evi1 Overexpression

Spleen cells from C57BL/6 mice that had succumbed to AML following transplantation with *Flt3*-ITD/*Npm1c*-transformed hematopoietic cells [38] were cultured in IMDM medium (Thermo Fisher Scientific, Waltham, MA, USA) containing 10% fetal bovine serum (Thermo Fisher Scientific), 1% L-glutamine (Thermo Fisher Scientific), 50 ng/mL mSCF (Peprotech, Hamburg, Germany), 10 ng/mL mIL-3 (Peprotech), and 10 ng/mL mIL-6 (BioLegend, San Diego, CA, USA). To generate Evi1^high^ and Evi1^low^ variants of *Flt3*-ITD/*Npm1c*-driven AML, these cells were transduced either with a vector encoding an epitope-tagged version of murine *Evi1* (pMYs_FLAG-*Evi1*_IRES_GFP, kindly provided by Dr. Takuro Nakamura, Cancer Institute of JFCR, Tokyo, Japan) or with empty vector as a control. In brief, vectors were transfected into Platinum-E cells, along with the ecotropic packaging plasmid psi2 (containing the *gag*, *pol*, and *env* genes) using a standard calcium chloride protocol. Virus-containing supernatants were harvested after 48–96 h, filtered (0.45 µm pore size), and supplemented with polybrene (4 μg/mL). Cells were spinoculated with retroviral supernatant for 60 min at 1300 rpm and 34 °C. The process was repeated with fresh retroviral supernatant after 24 and 48 h. Five days after the last transduction, *Flt3*-ITD/*Npm1*c_*Evi1* and *Flt3*-ITD/*Npm1*c_vec cells were sorted for GFP positivity and expanded in the above-described medium. For transplantation, 6–8-week-old female C57BL/6 recipient mice were sub-lethally irradiated (5 Gy). On the next day, mice were anesthetized by *i.p.* injection of 100 µL Ketasol/Rompun solution (18.5 mg/mL Ketasol (AniMedica, Senden, Germany), 1.5 mg/mL Rompun (Bayer, Leverkusen, Germany), and 0.9% sodium chloride (Braun, Kronberg, Germany)) followed by retro-orbital injection of 400,000 *Flt3*-ITD/*Npm1*c_*Evi1* or *Flt3*-ITD/*Npm1*c_vec cells. Mice were monitored for signs of disease (immotility, hunched posture, scrubby fur, loss of body weight), and sacrificed when terminally ill. Their BM and spleen cells were collected and vitally frozen. The GFP-positive fractions of these cells were considered as LCs, and are referred to as LC^*Flt3*-ITD/*Npm1c*_*Evi1*^ and LC^*Flt3*-ITD/*Npm1c*_vec^, respectively. For ex vivo experiments, cells were thawed and maintained in the medium described at the beginning of this chapter.

### 2.3. Drug Treatment, Cell Viability (Metabolic Activity), and Apoptosis Assays

For cell viability and apoptosis assays, BM LC^*Flt3*-ITD/*Npm1c*_vec^ and LC^*Flt3*-ITD/*Npm1c*_*Evi1*^ were seeded at 200 cells/µL and incubated with various concentrations of atRA (Sigma-Aldrich, St. Louis, MO, USA) or with solvent for 48 h. Metabolic activity as a proxy for cell viability was determined in white-walled 96-well-plates (Greiner Bio-One, Kremsmuenster, Austria) using the CellTiter-Glo^®^ Luminescent Cell Viability Assay (Promega, Madison, WI, USA). Luminescence was measured using the Varioskan LUX microplate reader with SkanIt Software for Microplate Readers RE, Version 5.0.0.42. (Thermo Fisher Scientific).

Annexin V assays were performed to quantify the proportions of apoptotic cells after drug treatment. Thus, cells were stained with 2 µL of Annexin V-APC (BD Biosciences, Franklin Lakes, NJ, USA) in 100 µL Annexin V binding buffer (10 mM HEPES (Sigma-Aldrich), pH 7.4, 140 mM NaCl (Carl Roth, Karlsruhe, Germany), and 2.5 mM CaCl_2_ (Sigma-Aldrich)) for 15 min at room temperature, and analyzed by flow cytometry (LSR Fortessa, BD Biosciences). Annexin V^−^ cells were classified as viable and Annexin V^+^ as apoptotic.

### 2.4. Determination of Myeloid Differentiation by Flow Cytometry

To analyze myeloid differentiation, BM cells from leukemic mice were treated with 1 µM atRA (Sigma-Aldrich) or the corresponding amount of solvent for 72 h. Afterwards, 500,000 cells per sample were washed once with PBS and stained with 1 µL of CD11b (clone M1/70, BioLegend) and Gr-1 (clone RB6-8C5, BioLegend) antibodies in 100 µL 2% fetal bovine serum/PBS for 30 min. Cells were washed again and analyzed by flow cytometry (LSR Fortessa, BD Biosciences). Analyses were restricted to leukemic cells by gating on the GFP-positive population.

### 2.5. Serial Replating Assays

For serial replating assays, BM LC^*Flt3*-ITD/*Npm1c*_vec^ and LC^*Flt3*-ITD/*Npm1c*_*Evi1*^ were treated with 1 µM atRA or the corresponding amount of solvent for 72 h. Thereafter, 3000 cells were plated per well of a six-well plate in MethoCult GF M3434 (Stemcell Technologies, Vancouver, Canada). Every seven days, the numbers of colonies were quantified, and 3000 cells were used for replating.

### 2.6. Quantitative RT-PCR

Total RNA was extracted using Trizol (Thermo Fisher Scientific) and reverse-transcribed using random hexamer primers (Thermo Fisher Scientific) and M-MLV reverse transcriptase (Thermo Fisher Scientific). Quantitative RT-PCR (qRT-PCR) was performed on a Step One Plus Real Time PCR system (Thermo Fisher Scientific) using GoTaq qPCR Master Mix (Promega) and the following primers: *Evi1* (fwd: 5′-CTCGAAGCCTTCAGGAACAC-3′, rev: 5′-AGCTTCAAGCGGGTCAGTTA-3′), *ß-2-microglobulin* (fwd: 5′-CCTTCAGCAAGGACTGGTCT-3′, rev: 5′-TGTCTCGATCCCAGTAGACG-3′). Assays were performed in triplicate, and *Evi1* expression was normalized to *ß-2-microglobulin* expression using the ΔΔC_T_ method [39].

### 2.7. Immunoblot Analysis

Preparation of protein lysates from spleen LCs, SDS-PAGE, transfer to PVDF membranes (Hybond-P; Amersham, Amersham, United Kingdom), and antibody incubations were performed using standard procedures. The following antibodies were used: anti-FLAG (clone M2; Sigma-Aldrich; 1:1000) and anti-GAPDH (clone 14C10; Cell Signaling Technologies, Danvers, MA, USA; 1:50,000). Blots were developed using SuperSignal West Femto or Pico Chemiluminescent Substrate (both from Thermo Fisher Scientific) and scanned using a ChemiDoc Touch Imaging System (Bio-Rad Laboratories, Hercules, CA, USA).

### 2.8. Statistical Analyses of Experimental Data

Significance of differences between multiple groups was determined by 2-way ANOVA followed by Bonferroni’s post hoc test. In serial replating assays, numbers of colonies were expressed relative to LC^*Flt3*-ITD/*Npm1c*_vec^ -atRA in each round of plating; significance was assessed using the one-sample t-test for comparisons with the control and Student’s t-test for other comparisons. The log-rank test was used to evaluate survival differences between groups of mice. Two-sided *p*-values < 0.05 were considered statistically significant. Analyses were performed using GraphPad Prism 6 (San Diego, CA, USA) software.

## 3. Results

### 3.1. Experimental Expression of Evi1 in Flt3-ITD/Npm1c-Driven Murine AML Decreases Disease Latency

**Figure 1:**
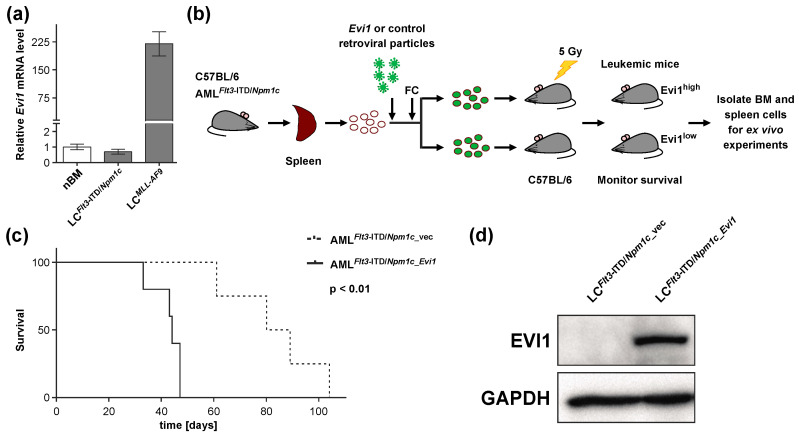
Experimental expression of *Evi1* decreases the latency of *Flt3*-ITD/*Npm1c*-driven murine AML. (**a**) Relative *Evi1* mRNA levels in normal murine bone marrow cells (nBM) and in leukemic cells from bone marrow (BM) of mice with *Flt3*-ITD/*Npm1c*- and *MLL-AF9*-driven AML (LC^*Flt3*-ITD/*Npm1c*^ and LC*^MLL-AF9^*, respectively; *n* = 3). (**b**) Schematic of experimental design. Spleen cells from mice terminally ill with *Flt3*-ITD/*Npm1c*-driven AML (AML^*Flt3*-ITD/*Npm1c*^) were transduced with pMSCV_FLAG-*Evi1*_IRES_GFP or with empty vector as a control. GFP-positive cells were sorted and transplanted into sub-lethally irradiated recipient mice. FC, flow cytometry; BM, bone marrow. (**c**) Kaplan–Meier plot of mice transplanted with *Flt3*-ITD/*Npm1c*_vec and *Flt3*-ITD/*Npm1c*_*Evi1* cells (400,000 cells/mouse). *n* = 4 (AML^*Flt3*-ITD/*Npm1c*_vec^ group), *n* = 5 (AML^*Flt3*-ITD/*Npm1c_Evi1*^ group). (**d**) Immunoblot analysis of FLAG-EVI1 expression in spleen LC^*Flt3*-ITD/*Npm1c_Evi1*^ and LC^*Flt3*-ITD/*Npm1c*_vec^ from terminally ill recipient mice. GAPDH was used as a loading control.

Human patients with cytogenetically normal AML may overexpress *EVI1* or not [12,13,40]. We therefore determined the expression of *Evi1* in *Flt3*-ITD/*Npm1*c-driven murine AML [38] using qRT-PCR. *Evi1* mRNA levels in BM from *Flt3*-ITD/*Npm1*c mice were comparable to those in normal mouse BM, and substantially lower than in BM from mice with *MLL-AF9*-driven AML (Figure 1a). Therefore, LCs from *Flt3*-ITD/*Npm1c* mice were transduced with a vector encoding an epitope-tagged version of murine *Evi1* (pMYs_FLAG-*Evi1*_IRES_GFP) or with empty vector as a control, and sorted for GFP positivity to yield *Flt3*-ITD/*Npm1*c_*Evi1* and *Flt3*-ITD/*Npm1*c_vec cells, respectively. These were transplanted into sub-lethally irradiated C57BL/6 recipient mice (400,000 cells/mouse; Figure 1b and Figure S1). In agreement with earlier observations [41,42], *Evi1* up-regulation dramatically decreased time to disease onset (Figure 1c; median survival, 84 and 44 days for recipients of *Flt3*-ITD/*NPM1*c_vec and *Flt3*-ITD/*NPM1*c_*Evi1* cells, respectively, *p* < 0.01). The presence of the EVI1 protein in LC^*Flt3*-ITD/*Npm1*c_*Evi1*^, but not LC^*Flt3*-ITD/*Npm1*c_vec^, obtained from terminally ill recipient mice was confirmed by immunoblot analysis using a FLAG antibody (Figure 1d).

### 3.2. atRA Reduces Viability and Stem Cell Related Properties in LCs from Flt3-ITD/Npm1c-Driven AML, but Experimental Expression of Evi1 Counteracts These Effects

**Figure 2:**
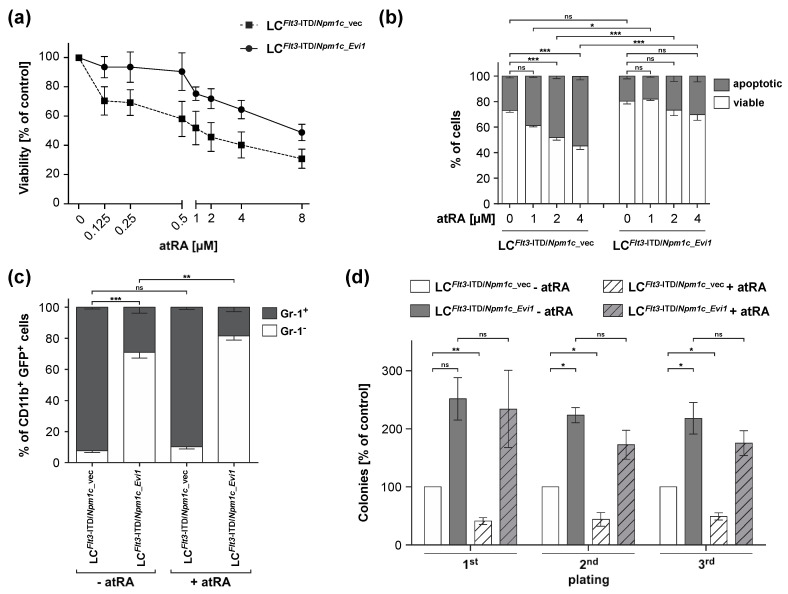
Experimental expression of *Evi1* counteracts the anti-leukemic and stem cell inhibitory effects of atRA in *Flt3*-ITD/*Npm1c*-driven murine AML. (**a**,**b**) Bone marrow LC^*Flt3*-ITD/*Npm1c*_vec^ and LC^*Flt3*-ITD/*Npm1c_Evi1*^ were treated with the indicated concentrations of atRA or with solvent for 48 h. (**a**) Cell viability was determined using metabolic activity as a proxy (Cell-Titer Glo^®^ assay). *n* = 4. (**b**) Apoptosis was determined through Annexin V staining followed by flow cytometry. *n* = 2–4. (**c**) Myeloid differentiation. Bone marrow cells from leukemic mice were treated with 1 µM atRA or the corresponding amount of solvent for 72 h, stained with CD11b and Gr-1 antibodies, and subjected to flow cytometry. Analyses were restricted to leukemia cells (LCs) by gating on GFP-positive cells. *n* = 3. (**d**) Serial replating activity. Bone marrow LC^*Flt3*-ITD/*Npm1c*_vec^ and LC^*Flt3*-ITD/*Npm1c_Evi1*^ were treated with 1 µM atRA or the corresponding amount of solvent for 72 h. A total of 3000 cells were plated per well of a six-well plate in MethoCult GF M3434. Every seven days, the numbers of colonies were quantified, and 3000 cells were re-plated. Numbers of colonies are expressed relative to those obtained with solvent-treated LC^*Flt3*-ITD/*Npm1c*_vec^ in each round of plating. *n* = 3. (**a**–**d**) *, *p* < 0.05; **, *p* < 0.01; ***, *p* < 0.001; ns, not significant.

To determine the effects of atRA, and its interactions with the expression of *Evi1*, on viability, apoptosis, differentiation, and stem cell-related properties of LCs from *Flt3*-ITD/*Npm1c*-driven AML, BM LC^*Flt3*-ITD/*Npm1*c_vec^ and LC^*Flt3*-ITD/*Npm1*c_*Evi1*^ were treated with atRA or solvent and subjected to appropriate assays. A three-day ex vivo incubation with atRA decreased the viability of LC^*Flt3*-ITD/*Npm1*c_vec^ in a dose-dependent manner, while experimental expression of *Evi1* reduced the sensitivity towards atRA in this assay (IC_50_, 1.12 µM for LC^*Flt3*-ITD/*Npm1*c_vec^ and 2.95 µM for LC^*Flt3*-ITD/*Npm1*c_*Evi1*^; Figure 2a). LC^*Flt3*-ITD/*Npm1*c_*Evi1*^ also exhibited a slightly lower rate of basal apoptosis than LC^*Flt3*-ITD/*Npm1*c_vec^, and had a strongly and significantly diminished response to the cell death-promoting effect of atRA (Figure 2b and Figure S2a). Further underscoring the increased aggressiveness of AML with *Evi1* overexpression, LC^*Flt3*-ITD/*Npm1*c_*Evi1*^ contained a much higher proportion of immature (Gr-1^−^) cells within the myeloid (CD11b^+^) compartment than LC^*Flt3*-ITD/*Npm1*c_vec^ (Figure 2c and Figure S2b). atRA slightly increased the proportion of immature myeloid cells among LC^*Flt3*-ITD/*Npm1*c_*Evi1*^ but had no significant effect on LC^*Flt3*-ITD/*Npm1*c_vec^ (Figure 2c and Figure S2b). Finally, *Evi1* overexpression increased the serial replating efficiency, an indicator of LSC activity, of *Flt3*-ITD/*Npm1c*-driven AML cells (Figure 2d and Figure S2c). Importantly, atRA reduced the replating ability of LC^*Flt3*-ITD/*Npm1*c_vec^, but had no significant effect on that of LC^*Flt3*-ITD/*Npm1*c*_Evi1*^ (Figure 2d and Figure S2c).

In summary, our results demonstrate anti-leukemic effects of atRA towards LCs and LSCs from *Flt3*-ITD/*Npm1c*-driven AML. These effects were counteracted by experimental expression of *Evi1*. These data confirm the earlier noted genetic and molecular complexity of the responsiveness of AML LSCs to atRA [27,37].

## 4. Discussion

atRA in combination with chemotherapy and, more recently, arsenic trioxide has greatly improved the outcome of APL [15,16,17]. In contrast, the success of atRA in non-APL AML has been limited so far [31]. Further, the sensitivity of certain molecularly or genetically defined AML subgroups (defined by *MN1* expression or *NPM1* mutations, respectively) was not confirmed in subsequent studies [20,27,29,30,31,32]. Nevertheless, atRA and other retinoids remain attractive options for the treatment of AML because of their low toxicity and abundant preclinical data suggesting their possible effectiveness in this disease [18,19,20,21,22,23,24,25,26,27,36]. The ongoing clinical interest in the use of atRA in AML is indicated by recent publications [43,44] and the fact that several trials are currently recruiting (www.clinicaltrials.gov).

Surprisingly, even though AML is well established as a stem cell-driven disease [33], to date very few studies have addressed the effect of atRA on AML LSCs. In *AML1-ETO*-expressing murine BM cells, atRA increased serial replating ability and led to the formation of larger and more immature colonies [21], suggesting that atRA promoted LSC activity on this genetic background. Similarly, atRA promoted leukemic stemness in an *MLL-AF9*-driven mouse model of AML in a manner dependent on the expression of *Evi1* [37]. *MLL* rearrangements are frequently associated with *EVI1* overexpression in human AML [45,46], and this is reflected in the corresponding mouse model [37,47]. In BM cells from *MLL-AF9* leukemic mice, atRA promoted the abundance and quiescence of an immunophenotypically defined LSC-enriched cell population and enhanced the activity of LSCs as determined by serial replating and in vivo limiting dilution assays. These effects of atRA were abolished by knock-down of *Evi1* [37]. Correspondingly, an RAR antagonist decreased LSC-related properties in an *Evi1*-dependent manner in vitro and in vivo, and prolonged survival of both of primary treated and secondary recipient mice with Evi1^high^, *MLL-AF9*-driven AML. Confirmatory results were obtained with both human AML cell lines and with primary AML samples [37].

In sharp contrast to the observations with the *AML1-ETO* and *MLL-AF9* models, atRA inhibited LSC activity in an AML mouse model driven by an *Flt3*-ITD in combination with a *Nup98-Hoxd13* fusion gene [36]. Treatment of primary mice with atRA alone or in combination with the tyrosine kinase inhibitor sorafenib prolonged time to disease onset in secondary recipients. Moreover, an in vivo limiting dilution assay with cells from the treated mice revealed ~9-fold, ~500-fold, and >12,000-fold reductions in LSC frequencies upon treatment with atRA, sorafenib, and atRA + sorafenib, respectively [36]. The present study corroborates the notion that the *Flt3*-ITD—which in this case was combined with an *Npm1* mutation in order to reveal its leukemogenic potential—renders AML LSCs sensitive to the inhibitory effects of atRA. Furthermore, we show that overexpression of *Evi1* abolishes the inhibitory effect of atRA on *Flt3*-ITD/*Npm1*c-bearing LSCs. Together, these data suggest that complex genetic and molecular interactions determine the response of AML LSCs to atRA. Further underscoring this, and in sharp contrast to its role in AML, on the background of the APL-typical *PML-RARA* fusion, the *Flt3*-ITD mutation reduced the inhibitory effect of atRA on the ability to initiate leukemia in secondary recipients [48].

The molecular and genetic complexity determining the atRA responsiveness of AML LSCs may explain, to some extent, why it has been difficult to identify patient groups benefitting from atRA in non-APL AML. Other aspects that warrant consideration are the identity of the drugs used in conjunction with atRA, as well as the timing and duration of treatment. Different trials assessing the efficacy of atRA in AML were based on different chemotherapeutics. atRA was initiated at different time points relative to the start of chemotherapy, and was included in the maintenance therapy in some studies but not others [31]. Prolonged administration of atRA may be of particular importance in cases where atRA inhibits LSCs. On the other hand, in AML with aberrations rendering their LSCs positively responsive to atRA, there might even be a therapeutic role for RAR antagonists, even though the effects of atRA on normal HSCs [49,50,51,52] need to be carefully considered in this context. Other aspects concerning the identity of the retinoids themselves also warrant attention. Certain synthetic retinoids are resistant to degradation by CYP26 [53], which may increase their clinical effectiveness. Moreover, atRA mediates its effects through different nuclear receptor isoforms that can be targeted by specific agonists and antagonists. Among these isoforms, RARA and RARG were reported to promote myeloid differentiation [21,51,53] and HSC activity [51], respectively. The identity of the isoforms mediating the activity of atRA towards LSCs has been queried only in the context of *AML1-ETO*, with complex results: only the combination of a RARA and a RARG agonist reproduced the LSC-promoting effects of atRA [21].

Further research is needed to carefully dissect which patient subgroups may benefit from which type of retinoid, and how retinoids can be combined with other drugs to maximize anti-leukemic effects. An enhanced understanding of the effects of retinoids on LSCs may ultimately facilitate the development of precision medicine-based retinoid therapy for certain subgroups of AML.

## Supplementary Materials

Supplementary materials can be found at https://www.mdpi.com/2227-9059/8/10/385/s1.
