# Supplementary material for: Evi1 Counteracts Anti-Leukemic and Stem Cell Inhibitory Effects of All-Trans Retinoic Acid on Flt3-ITD/Npm1c-Driven Acute Myeloid Leukemia Cells
Source: Biomedicines. 2020 Sep 28;8(10):385. doi: 10.3390/biomedicines8100385 (PMC7600968; doi:10.3390/biomedicines8100385)
Supplement: Supplementary file 1 [file biomedicines-08-00385-s001.pdf]

Supplemental Figures to manuscript "*Evi1* counteracts anti-leukemic and stem cell inhibitory effects of *all-trans* retinoic acid on *Flt3*-ITD/*Npm1c*-driven acute myeloid leukemia cells" by Nguyen et al.

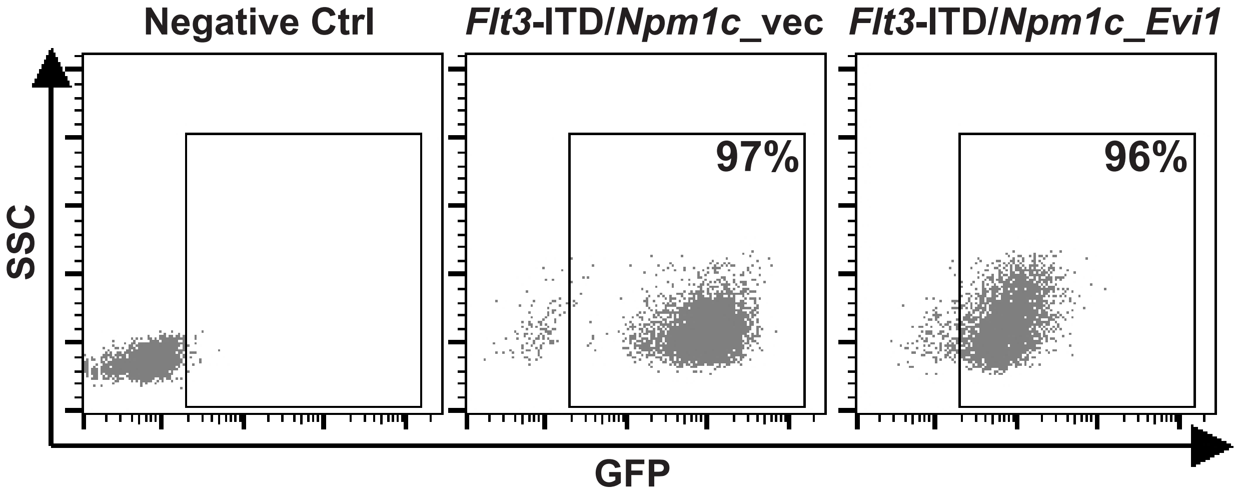

Figure S1. Proportion of GFP positive cells among LC$^{Flt3\text{-ITD}/Npm1c}$ transduced with empty vector (*Flt3*-ITD/*Npm1*c_vec) or with pMSCV_FLAG-*Evi1*_IRES_GFP (*Flt3*-ITD/*Npm1*c_*Evi1*). Representative flow cytometric analysis performed after sorting. SSC, side scatter. As negative control (Ctrl), a cell line established from primary murine AML cells (RN2) was used.

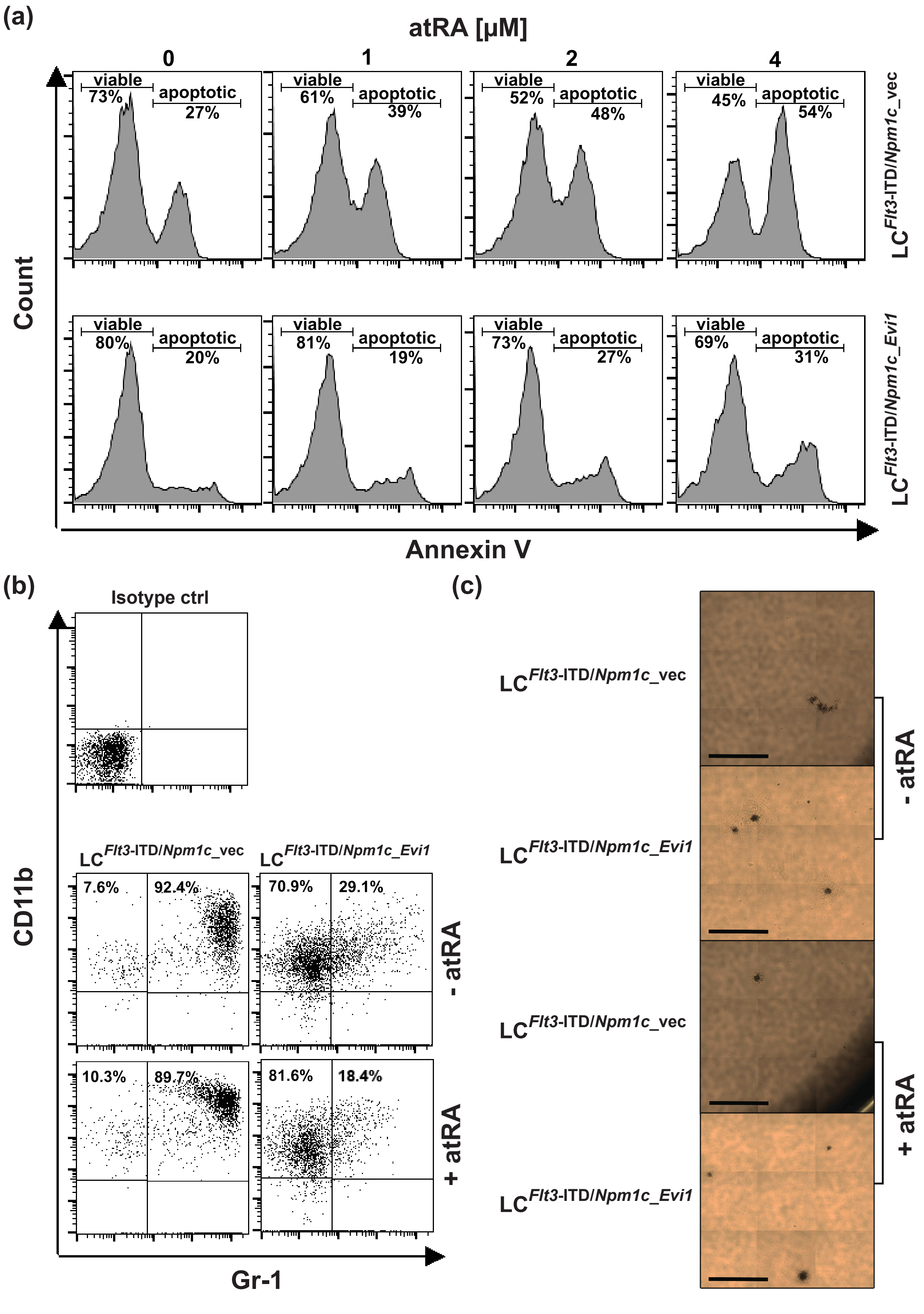
(a)
atRA [µM]
0
1
2
4
viable
73%
apoptotic
27%
viable
61%
apoptotic
39%
viable
52%
apoptotic
48%
viable
45%
apoptotic
54%
LCFlt3-ITD/Npm1c_vec
viable
80%
apoptotic
20%
viable
81%
apoptotic
19%
viable
73%
apoptotic
27%
viable
69%
apoptotic
31%
LCFlt3-ITD/Npm1c_Evi1
Count
Annexin V
(b)
Isotype ctrl
LCFlt3-ITD/Npm1c_vec
LCFlt3-ITD/Npm1c_Evi1
7.6%
92.4%
70.9%
29.1%
- atRA
10.3%
89.7%
81.6%
18.4%
+ atRA
CD11b
Gr-1
(c)
LCFlt3-ITD/Npm1c_vec
LCFlt3-ITD/Npm1c_Evi1
- atRA
LCFlt3-ITD/Npm1c_vec
LCFlt3-ITD/Npm1c_Evi1
+ atRA

Figure S2. Experimental expression of *Evi1* counteracts the anti-leukemic and stem cell inhibitory effects of atRA on *Flt3*-ITD/*Npm1c* driven murine AML cells. Bone marrow $LC^{Flt3\text{-ITD}/Npm1c\_\text{vec}}$ and $LC^{Flt3\text{-ITD}/Npm1c\_Evi1}$ were treated with the indicated concentrations of atRA or with solvent for 48 h (a), or with 1 µM atRA or the corresponding amount of solvent for 72 h (b, c). (a) Apoptosis. Representative flow cytometric analysis of Annexin V stained cells. Annexin V negative and positive cells were considered as viable and apoptotic, respectively. (b) Myeloid differentiation. Representative flow cytometric analyses of $Gr1^+$ cells among $CD11b^+$ GFP^+ cells. Pre-gating on the GFP positive population restricted the analysis to leukemic cells. Cut-offs for marker positivity were determined using isotype control antibodies. (c) Serial replating assay. Representative images of colonies from second plating. Scale bars, 2mm. Images were taken using TissueFAXS i PLUS (TissueGnostics, Vienna, Austria) and TissueFAXS v.4.2 software.
